# Donor lymphocyte infusion for prevention of relapse after unmanipulated haploidentical PBSCT for very high-risk hematologic malignancies

**DOI:** 10.1007/s00277-018-3482-7

**Published:** 2018-08-24

**Authors:** Xiao-Ning Gao, Ji Lin, Shu-Hong Wang, Wen-Rong Huang, Fei Li, Hong-Hua Li, Jing Chen, Li-Jun Wang, Chun-Ji Gao, Li Yu, Dai-Hong Liu

**Affiliations:** 1 Department of Hematology, Chinese PLA General Hospital, Medical School of Chinese PLA, 28 Fuxing Road, Beijing, 100853 China; 2 Institute of Basic Medicine, Chinese PLA General Hospital, Medical School of Chinese PLA, 28 Fuxing Road, Beijing, 100853 China

**Keywords:** Donor lymphocyte infusion, Stem cell transplantation, Peripheral blood, Graft-versus-host disease, Relapse

## Abstract

Unmanipulated haploidentical peripheral blood stem cell transplantation (haplo-PBSCT) has been an established treatment to cure high-risk leukemia/lymphoma. Relapse is the main cause of treatment failure for patients with relapsed/refractory disease or with very high-risk gene mutations such as *TP53*, *TET2*, and *DNMT3a*. In this study, we aimed to establish the tolerance and efficacy of prophylactic donor lymphocyte infusion (DLI) with G-CSF-primed peripheral blood progenitors for prevention of relapse in these very high-risk patients after haplo-PBSCT. The prophylactic DLI was given at a median of 77 days after transplantation in 31 of 45 consecutive patients with very high-risk leukemia/lymphoma. The median dose of CD3^+^ cells for infusion was 1.8 × 10^7^/kg. The 100-day incidences of acute graft-versus-host disease (GVHD) grades 2–4 and 3–4 after DLI were 55.3% and 10.2%. The 2-year incidences of chronic GVHD and severe chronic GVHD were 52.0% and 18.2%. The 2-year incidences of non-relapse mortality and relapse were 33.1% and 32.5%. The 2-year probabilities of overall survival and relapse-free survival were 40.1% and 31.9%. Poor-risk gene mutations (*p* = 0.029), disease in non-remission status prior to transplantation (*p* = 0.005), and donors older than 40 years of age (*p* = 0.043) were associated with relapse after DLI. In multivariate analysis, disease in non-remission status prior to transplantation was an independent risk factor of relapse (hazard ratio = 4.079; *p* = 0.035). These data showed the feasibility of the prophylactic DLI in the haplo-PBSCT setting and the anti-leukemic efficacy in very high-risk leukemia/lymphoma.

**Electronic supplementary material:**

The online version of this article (10.1007/s00277-018-3482-7) contains supplementary material, which is available to authorized users.

## Introduction

The prognosis of acute leukemia or high-grade lymphoma in relapsed/refractory status is dismal [1, 2]. More recently, gene mutations, such as *TP53*, *DNA-methyltransferase-3a* (*DNMT3a*) or *ten-eleven translocation-2* (*TET2*) mutation, have been identified by the next-generation sequencing technique as very high-risk molecular markers for acute leukemia with low rate of remission and short survival [3–5]. Though the targeted therapy developed in recent years have resulted in an increased rate of remission and improved survival in a small subset of these very high-risk patients, allogeneic hematopoietic stem cell transplantation (allo-HCT) is still the most effective way for cures. However, the recurrence of the underlying disease after transplantation remains the leading cause of treatment failure. The rate of relapse of the leukemia/lymphoma that was in refractory/relapsed status prior to transplantation and those with very high-risk gene mutations ranged from 50 to 60% and the long-term relapse-free survival (RFS) was less than 30% after allo-HCT [6, 7].

A reasonable approach to improve the survival of the very high-risk leukemia/lymphoma is to explore prophylactic strategies after transplantation to reduce the relapse rate. Donor lymphocyte infusion (DLI) has been proved to be effective to stimulate graft-versus-leukemia (GVL) reaction in patients with minimal residual disease (MRD) or hematologic relapse after transplantation [8, 9]. However, the utility of DLI is limited by the toxicity of fatal GVHD or pancytopenia and subsequent infections resulting in an increase in non-relapse mortality (NRM). We have shown that using granulocyte colony-stimulating factor (G-CSF)-primed peripheral blood cells (G-PB) for DLI with substitution of the steady-state lymphocytes could reduce the DLI-associated fatal GVHD without counteracting its GVL effect [10]. Reduced GVHD with improved RFS has been established in the unmanipulated haploidentical-HCT with bone marrow and peripheral blood (BM + PB) as grafts for therapeutic, preemptive and prophylactic use of DLI [11–13]. However, all of these studies were in the setting of G-CSF-primed BM + PB as the graft source. It is well known that the components of the graft have an influence on the development of GVHD. In general, the peripheral blood stem cell transplantation (PBSCT) could be associated with a higher incidence of GVHD compared with allo-HCT with BM as grafts. The safety and efficacy of prophylactic G-PB DLI in the setting of unmanipulated haploidentical PBSCT (haplo-PBSCT) have not been determined. In this study, we used prophylactic G-PB DLI for the very high-risk leukemia/lymphoma patients after haplo-PBSCT with modifications intending to alleviate DLI-associated GVHD. The modifications included that the dose of infused CD3^+^ cells was reduced to less than 2 × 10^7^/kg (BM + PB setting, 4 × 10^7^/kg), and the time interval between haplo-PBSCT and DLI was postponed to 60~90 days (BM + PB setting, 45~60 days). The data of tolerance and efficacy of prophylactic G-PB DLI in 31 patients with very high-risk features was presented.

## Methods

### Study design

We did a retrospective, observational cohort study of a total of 45 consecutive patients with very high-risk leukemia/lymphoma who underwent haplo-PBSCT in our center between March 1, 2014 and January 31, 2018 (Supplementary Table 1). All of the patients enrolled in this study have not been reported in previous study. They were given a haplo-PBSCT because of lacking matched sibling or unrelated donor. The very high-risk features were defined by the following criteria: (i) disease in non-remission (NR) status including primary induction failure or relapsed, (ii) acute leukemia achieving first complete remission (CR) with > 2 cycles of induction of chemotherapy, (iii) leukemia with *TET2*, *DNMT3a*, or *TP53* mutation, and (iv) leukemia with normal cytogenetics and *FLT3-ITD* or chronic myelogenous leukemia (CML) with *BCR-ABL* T315I mutation (due to unavailability of targeted therapy). The study was approved by the Ethics Committee of Chinese PLA General Hospital, and signed informed consents were obtained from all patients prior to transplantation in accordance with principles of Declaration of Helsinki.

The patients with very high-risk features with full donor chimerism and negative MRD would receive prophylactic DLI at days 60 to 90 after haplo-PBSCT if no GVHD developed. If GVHD occurred before day + 60, DLI was delayed to 8 weeks after disappearance of symptoms and signs of GVHD. The patients with infection before day + 60 would receive prophylactic DLI after 4 weeks of disappearance of symptoms and stable improvement of the signs of infection. The G-PB cells infused were thawed from the cryopreserved product at the time of graft collection. The number of CD3^+^ cells scheduled for infusion was 2 × 10^7^/kg at a single dose. Cyclosporine A (CsA) started after transplant was not mandatory to stop prior to DLI. CsA was given at 2 mg/kg b.i.d from days − 3 to + 90, then be tapered at 33% per month to be discontinued on days + 150 to + 180 unless GVHD developed. If the patients received prophylactic DLI before day + 90, CsA was used 6 weeks (though concentration 150–250 ng/ml) after DLI for prophylaxis of DLI-associated GVHD, and then tapered and discontinued within 2 weeks except GVHD was present. If GVHD occurred before day + 90, DLI would be delayed to 8 weeks after GVHD was well controlled and CsA would be continued until 6 weeks after DLI, and then tapered over 2 weeks except GVHD occurred. Patients with positive MRD or hematologic relapse before day + 60 received chemotherapy followed by preemptive or therapeutic DLI and were not evaluated in this study.

### Transplantation procedure

For patients without organ dysfunction, the busulfan (Bu)-based myeloablative conditioning regimen was used, which consists of Bu (3.2 mg/kg, days − 10 to − 8), carmustine (250 mg/m^2^, day − 7), cytarabine (4 g/m^2^, days − 6 to − 5), and cyclophosphamide (Cy; 50 mg/kg, days − 4 to − 3). For patients with organ dysfunction during chemotherapy, Cy was substituted with fludarabine (30 mg/m^2^, days − 7 to − 3) due to organ dysfunction during chemotherapy. For patients with refractory B cell acute lymphoblastic leukemia, TBI-Cy regimen was used, which was consists of total body irradiation (8 Gy, day − 7), cytarabine (4 g/m^2^, days − 6 to − 5), and Cy (60 mg/kg, days − 4 to − 3). Anti-thymoglobuline (rabbit; Genzyme Europe BV; 2.5 mg/kg/d, days − 5 to − 2) was given to all recipients for prophylaxis of GVHD in addition to the routine regimen (CsA, mycophenolate mofetil, and short-term MTX). All recipients received G-PB as a source of graft. The supportive therapy was done as previously described [14].

### Definitions and statistical analyses

All patients alive were followed-up from the date of graft infusion to March 31, 2018. Days prior to graft infusion was documented with “−” and those after graft infusion with “+.” Relapse was defined as hematologic recurrence of malignancies after HCT. GVHD and post-DLI GVHD were assessed as previously defined [15, 16]. NRM was defined as death from any cause without relapse. Cumulative incidences (CIs) of GVHD, viral reactivations, relapse, and NRM were analyzed in a competing risk framework using Gray’s method [17, 18]. Probabilities of RFS and OS were calculated with 95% confidence intervals using Kaplan-Meier estimates. Factors for univariate analysis of risk for GVHD, relapse, NRM, OS, or RFS were patient’s age (< 40 years vs. ≥ 40 years), donor’s age (< 40 years vs. ≥ 40 years), poor-risk gene mutations (no vs. yes), disease status at HCT (CR vs. NR), and the interval from diagnosis to transplant (< 6 months vs. ≥ 6 months). All variables associated with a *p* < 0.15 by univariate analyses were included into the multivariate analysis. Statistical analyses were performed using R statistical software with cmprsk package of (www.r-project.org), Stata 14.0 software, and SPSS 20.0 software.

## Results

### Implementation of the prophylactic DLI

Of the 45 patients with very high-risk features, 31 received the prophylactic DLI (Table 1 and supplementary Table 2). The median time of DLI was 77 (45–240) days after transplantation. The reasons for delay of DLI were GVHD (*n* = 12), pancytopenia (*n* = 3), and renal dysfunction due to hemorrhagic cystitis (*n* = 1). Patient 23 received prophylactic DLI on day + 240 after HCT because of GVHD and cytomegalovirus-associated pancytopenia. The median doses of mononuclear cells and CD3^+^ cells for infusion were 0.6 (0.2–1.3) × 10^8^/kg and 1.8 (0.4–6.9) × 10^7^/kg, respectively. No onset of DLI-associated pancytopenia was documented in these patients.

**Table 1:** Characteristics of prophylactic DLI recipients and donors

Variable	Number	Percentage
Age of patient at transplantation (years)		
Median (range)	34 (18–57)	
< 40/ ≥ 40	20/11	64.5%/35.5%
Gender		
Male/female	18/13	58.1%/41.9%
WBC count at diagnosis^a^		
< 30 × 10^9^/L/ ≥ 30 × 10^9^/L	21/7	67.7%/22.6%
Diagnosis		
AML	21	67.7%
ALL/LBL	5	15.6%
CML	2	6.5%
NHL	2	6.5%
PCL	1	3.2%
Disease status at transplantation		
Primary induction failure	6	19.4%
Relapse untreated or refractory to reinduction CT	4	12.5%
CR1	19	61.3%
CML in CP1	2	6.5%
High-risk gene mutations^b^		
No/yes	11/20	35.5%/64.5%
High-risk cytogenetics^a, c^		
No/yes	27/2	87.1%/6.5%
Conditioning regimen		
Bu/Cy	25	80.6%
Bu/flu	4	12.9%
Cy/TBI	2	6.5%
Time from diagnosis to transplantation (days)		
Median (range)	173 (84–2737)	
Age of donor (years)		
Median (range)	28 (17–55)	
< 40/ ≥ 40	23/8	74.2%
HLA matched loci		25.8%
5/10	26	83.9%
6/10	3	9.4%
7/10	1	3.2%
8/10	1	3.2%
Donor-recipient ABO match		
Match	13	40.6%
Major mismatch	8	25.0%
Minor mismatch	9	29.0%
Bidirectional mismatch	1	3.2%
Donor-recipient gender match		
Female to male	7	22.6%
Female to female	4	12.9%
Male to female	9	29.0%
Male to male	11	35.5%
Graft ( × 10^8^/kg)		
MNCs, median (range)	9.7 (5.2–22.9)	
CD34^+^, median (range)	4.2 (1.9–7.6)	

*ALL*, acute lymphoblastic leukemia; *AML*, acute myeloid leukemia; *CML*, chronic myeloid leukemia; *CP*, chronic phase; *CR*, complete remission; *CT*, chemotherapy; *LBL*, lymphoblastic leukemia/lymphoma; *MNCs*, mononuclear cells; *NHL*, non-Hodgkin lymphoma; *PCL*, plasma cell leukemia; *WBC*, white blood cell

^a^Information is not available in some cases

^b^High-risk gene mutations indicate *TET2*, *DNMT3a*, *TP53*, *FLT3-ITD*, and *BCR-ABL* T315I mutations

^c^High-risk cytogenetics was defined as: (i) ALL with hypodiploidy (< 44 chromosomes), *t* (4;11), (9;22), or *t* (1;19); (ii) AML with monosomy 5, monosomy 7, 11q23, inv.(3), *t* (3;3), or *t* (9;22); (iii) Disease with complex karyotype (≥ 3 chromosomal abnormalities) or − 17

A total of 14 patients with very high-risk features did not receive prophylactic DLI due to early relapse (*n* = 8), intermittent GVHD or infection and subsequent pancytopenia, and poor general condition (*n* = 6). Of the eight patients with early relapse, five had disease recurrence before day + 60, and three developed GVHD and relapsed during the treatment of GVHD. The median time of relapse for these patients was 84.5 (36–168) days after haplo-PBSCT. Of the 14 patients without receiving scheduled prophylactic DLI treatment, eight died of relapse, two died of GVHD and four were still alive at the time of analysis. These four living patients recovered from GVHD and infections at 6 months post-transplantation with negative MRD, and they did not receive prophylactic DLI for tardy recovery from pancytopenia or poor general condition.

### GVHD after the prophylactic DLI

**Fig. 1:**
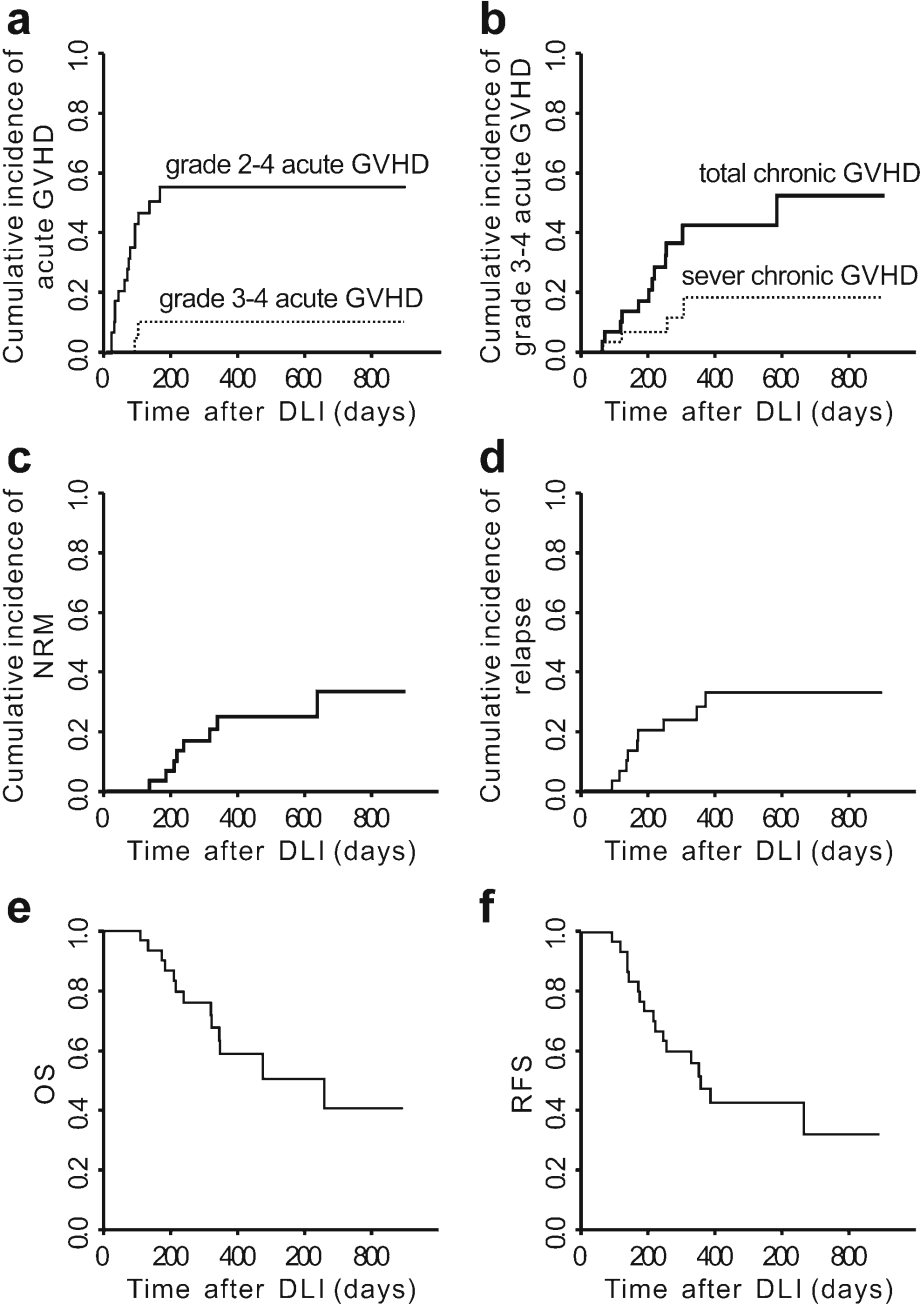
Transplantation outcomes of prophylactic DLI recipients. GVHD, graft-versus-host disease; NRM, non-relapse mortality; OS, overall survival; RFS, relapse-free survival

Eighteen (58.1%) of the 31 patients who received prophylactic DLI developed acute GVHD grades 1–4 at a median of 64 (11–165) days after prophylactic DLI (grade 1 in 3 cases, grade 2 in 13 cases, grade 4 in 2 cases). Seven of the 13 patients with acute GVHD grade 2 died. Of these seven patients, one died of interstitial pneumonia, one died of intracranial hemorrhage, one died of disease relapse and three died of GVHD. Of the two patients with acute GVHD grade 4, one was cured and survived free of relapse and another one died of disease relapse. The CIs of acute GVHD grades 2–4 and 3–4 were 55.3% (95% CI 33.7–72.4%) and 10.2% (95% CI 1.6–28.6%) at 100 days after DLI, respectively (Fig. 1a). No factors tested significantly correlated with the risk of occurrence of acute GVHD in univariate analysis (Table 2).

**Table 2:** Univariate analysis for the risk factors of transplant outcomes in prophylactic DLI recipients

Variable	Grades II–IV acute GVHD		Grades III–IV acute GVHD		Chronic GVHD		NRM		Relapse		RFS		OS	
	% (95% CI)	*p* value	% (95% CI)	*p* value	% (95% CI)	*p* value	% (95% CI)	*p* value	% (95% CI)	*p* value	% (95% CI)	*p* value	% (95% CI)	*p* value
Age														
< 40 years old	53.6 (26.2–74.8)	0.505	15.3 (2.1–40.1)	0.312	55.2 (16.4–82.2)	0.942	5.6 (0.3–23.3)	0.018	40.6 (17.2–63.0)	0.214	35.9 (8.2–65.7)	0.409	44.3 (12.1–73.1)	0.213
≥ 40 years old	56.8 (19.9–82.1)		0		41.7 (13.5–68.2)		55.2 (18.6–81.1)		20.5 (1.9–53.1)		24.3 (4.1–53.4)		36.5 (10.2–64.0)	
Poor-risk gene mutations														
No	50.0 (15.7–77.2)	0.635	13.0 (0.4–46.3)	0.838	54.6 (18.2–80.6)	0.391	18.2 (2.3–46.2)	0.345	54.6 (19.9–79.6)	0.029	27.3 (6.5–53.9)	0.131	30.9 (10.6–67.3)	0.600
Yes	58.6 (28.7–79.6)		8.6 (0.4–33.7)		43.1 (14.0–69.8)		29.9 (9.9–53.4)		18.0 (3.9–40.5)		26.1 (1.8–63.6)		31.5 (1.8–71.7)	
Disease status at transplantation														
CR	55.9 (30.1–75.5)	0.889	6.8 (0.4–27.5)	0.526	40.8 (16.2–64.4)	0.346	49.1 (11.0–79.5)	0.194	14.3 (3.4–32.6)	0.005	36.7 (8.6–66.3)	0.130	47.5 (16.2–73.7)	0.371
NR	54.2 (10.6–84.6)		17.9 (0.3–60.6)		48.2 (10.2–79.1)		11.1 (0.4–41.7)		74.1 (17.6–95.0)		14.8 (0.8–46.8)		23.3 (1.3–61.6)	
Donor’s age														
< 40 years old	60.2 (33.3–79.1)	0.362	6.9 (0.4–28.0)	0.397	52.9 (20.1–77.7)	0.577	43.2 (16.4–67.7)	0.131	24.0 (8.1–44.4)	0.043	32.8 (11.2–56.7)	0.493	35.6 (11.3–61.2)	0.778
≥ 40 years old	43.8 (7.7–76.6)		17.5 (0.4–57.1)		14.6 (0.4–50.9)		0		41.7 (7.2–74.7)		43.8 (10.1–74.2)		65.6 (15.7–90.9)	
Time from diagnosis to transplantation														
< 6 months	63.3 (29.0–84.5)	0.352	10.7 (0.4–40.0)	0.922	53.8 (11.2–83.9)	0.827	34.2 (11.2–59.1)	0.531	19.4 (4.3–42.4)	0.145	46.4 (20.4–69.0)	0.415	45.0 (19.2–68.0)	0.492
≥ 6 months	49.0 (18.1–74.3)		10.6 (0.4–39.6)		48.8 (16.2–75.4)		32.3 (3.0–69.4)		49.7 (16.5–76.3)		18.0 (1.3–50.9)		20.9 (1.0–58.6)	

*CR*, complete remission; *NR*, non-remission; *GVHD*, graft-versus-host disease; *NRM*, non-relapse mortality; *OS*, overall survival; *RFS*, relapse-free survival*; CI,* confidence interval

Chronic GVHD occurred in 12 (38.7%; mild in 3 cases, moderate in 5 cases, severe in 4 cases) patients. The median time of the onset of chronic GVHD was 205 (60–582) days after DLI. Nine patients with chronic GVHD had previous acute GVHD after DLI. The 6-month, 1-year, and 2-year CIs of chronic GVHD were 20.6% (95% CI 8.1–37.0%), 42.1% (95% CI 21.9–61.2%), and 52.0% (95% CI 24.6–73.7%), respectively (Fig. 1c). The 6-month, 1-year, and 2-year CIs of severe chronic GVHD were 6.7% (95% CI 1.1%–19.5%), 18.2% (95% CI 4.7–38.6%), and 18.2% (95% CI 4.7–38.6%), respectively (Fig. 1b). No factors tested significantly correlated with the risk of occurrence of chronic GVHD in univariate analysis (Table 2).

### NRM and relapse of the prophylactic DLI recipients

A total of eight patients (25.8%) died of non-relapse complications. Of these eight patients, five died of GVHD, two died of pneumonia and respiratory failure, and one with poor engraftment died of intracranial hemorrhage. No pathogens were recorded for the two patients died of pneumonia. The 6-month, 1-year, and 2-year CIs of NRM were 6.7% (95%CI 1.1–19.5%), 24.7% (95%CI 10.5–42.0%), and 33.1% (95%CI 13.1–54.9%), respectively (Fig. 1c). The cumulative risk of NRM after prophylactic DLI was higher in the patients older than 40 years of age as compared with those younger than 40 years of age (*p* = 0.015; Table 2). A total of 6/12 patients older than 40 years of age died of non-relapse complications. Of the six patients, four died of GVHD and two died of pneumonia.

Nine patients (29.0%) relapsed at a median of 87 (11–332) days after prophylactic DLI and 209 (87–656) days after HCT. The 6-month, 1-year, and 2-year CIs of relapse after HCT were 19.9% (95% CI 7.9–35.8%), 32.5% (95% CI 15.5–50.7%), and 32.5% (95% CI 15.5–50.7%), respectively (Fig. 1d). Poor-risk gene mutations (*p* = 0.029), NR status prior to HCT (*p* = 0.005), and donors older than 40 years of age (*p* = 0.043) correlated with a higher risk of relapse in univariate analyses (Table 2). In multivariate analysis, disease in NR status prior to HCT had the highest significant impact on relapse (hazard ratio = 4.079; *p* = 0.035; Table 3). Of the nine patients with relapse after prophylactic DLI, six were in NR status before transplantation. The median time of relapse for the six patients with pre-HCT NR disease was 114 (11–332) days after prophylactic DLI and 208 (87–379) days after transplantation.

**Table 3:** Multivariate analysis for the risk factors of relapse in prophylactic DLI recipients

Variable	HR	95% CI	*p* value
High-risk gene mutations			
No	1		
Yes	0.499	0.136–1.830	0.300
Disease status at transplantation			
CR	1		
NR	4.079	1.105–15.060	0.035
Donor’s age			
< 40 years old	1		
≥ 40 years old	3.102	0.884–10.890	0.077
Time from diagnosis to transplantation			
< 6 months	1		
≥ 6 months	1.800	0.545–5.940	0.330

CI, confidence interval; CR, complete remission; HR, hazard ratio; NR, non-remission

### Survival and the quality of life of the prophylactic DLI recipients

Median follow-up after haplo-PBSCT among surviving prophylactic DLI recipients was 383 (111–1174) days. At the time of analysis, 14/31 (45.2%) prophylactic DLI recipients were still alive in CR at a median of 274 (41–1129) days post-DLI. The Kaplan–Meier estimates for OS from transplantation at 1 and 2 years were 58.5% (95% CI 37.2–74.8%) and 40.1% (95% CI 16.3–63.1%; Fig. 1e). Estimated RFS from transplantation at 1 and 2 years was 47.3% (95% CI 28.0–64.4%) and 31.9% (95% CI 11.7–54.5%; Fig. 1f). The quality of life in prophylactic DLI recipients who survived without relapse was measured with Karnofsky performance scores. Twelve patients were 90–100, one was 80 and one was 50 due to chronic GVHD. No factors tested significantly correlated with OS or RFS in univariate analysis (Table 2).

## Discussion

The recurrence of disease is the primary cause of treatment failure and mortality after allo-HCT for patients with very high-risk hematologic malignancies. Prophylactic DLI has been proved effective for treatment, intervention, or prophylaxis of relapse when targeted therapy is lacking post-transplantation. Here, we showed the feasibility of G-PB DLI in the unmanipulated haplo-PBSCT setting with G-CSF primed PB as the graft source for prophylaxis of relapse in the very high-risk patients.

In previous study of transplantation with BM or with BM combined with PBSC as graft, we have shown that the incidence of severe GVHD after G-PB DLI was less compared with that after traditional DLI with steady-state lymphocytes [19]. In subsequent series of studies, the safety and efficacy of G-PB DLI were demonstrated in the treatment of relapse, preemptive therapy for MRD-positive patients or prophylaxis before relapse occurs after unmanipulated haploidentical HCT with PB + BM as grafts. It was reported that in 56 patients (29 after haplo-HCT) who were MRD-positive and received G-PB DLI, the incidence of acute GVHD grades 2–4 was 27.9% and that of chronic GVHD was 42.9% which were similar to those in the MRD negative patients (30.2 and 38.8%) who did not receive the intervention [20]. The use of G-PB DLI was based on the laboratory findings that G-CSF-priming could induce hypo-responsiveness of T cells for polarization from Th1 to Th2 and downregulation of the CD28/B7 pathway [10, 21] and augment NK-T cell dependent CD8^+^ cytotoxicity which might enhance GVL without GVHD [22]. Further, there was no less activity with G-CSF-primed as compared to untreated T cells [23]. Nevertheless, the evidence for G-CSF-priming retaining GVL activity is sparse. In the current transplantation setting without in vitro T cell depletion, there is little evidence that NK cells could replace the hindered T cells. Therefore, the delay of DLI for 60 days might be the most important reason for its tolerance, as the cytokine storm caused by myeloablative conditioning was over.

It has been shown that G-PB DLI with immunosupressants prophylaxis more than 6 weeks were associated with a lower incidence of acute GVHD grades 3–4. Further, DLI-associated acute GVHD grades 3–4 was the only risk factor for OS and NRM but not for relapse after DLI [11]. Therefore, in the current study, CsA was reduced and discontinued after the 6-week prophylaxis if no GVHD occurred. PBSCT with HLA-identical sibling donors was considered to be associated with an increased incidence of chronic GVHD compared with allo-HCT with BM [24]. Therefore, we postponed the timing for DLI and reduced the dose of infused CD3+ cells concerning the potential higher incidence of GVHD in the haplo-PBSCT setting. The incidences of DLI-associated acute GVHD grades 2–4, 3–4, chronic GVHD, and NRM in our study were 55.3%, 10.2%, 52.0,%, and 33.1%, respectively. In the patients who received haplo-PBSCT in our unit before January of 2015 [14], the incidences of acute GVHD grades 2–4, 3–4, chronic GVHD, and NRM were 36.1%, 14.5%, 38.4%, and 24.0%, respectively. Though the incidences of GVHD between these two cohort studies should not be compared directly, it seemed that G-PB DLI did not result in an intolerable toxicity in terms of GVHD and NRM. In addition, the tolerance of this procedure might be related to the following mastery of contraindications for prophylactic DLI: (1) the elimination of the patients with intermittent GVHD and (2) all prophylactic DLI was given after stable response of treatment of the previous GVHD.

An optimal timing of prophylactic DLI should be in a balance between GVL and GVHD because increasing the time interval between transplantation and DLI will lead to a decrease in the risk of DLI-associated toxicity but an increase in the likelihood of relapse. Because the median time to post-allo-HCT relapse or progression was 2 to 3 months for adult patients with high-risk acute myeloid leukemia and T cell leukemia/lymphoma [25, 26], it is reasonable to administrate the prophylactic strategy before day + 90 after HCT. The median time of the occurrence of acute GVHD in the unmanipulated haplo-PBSCT was + 30~ + 60 days after graft infusion. That means the prophylactic DLI candidates would face superimposed risk of GVHD if the time of DLI is before day + 60. Even though several patients, at the beginning stage of the current study, had received DLI before day + 60, it is reasonable and feasible to give the prophylactic DLI after day + 60 if no intermittent GVHD occurred or GVHD was stably controlled.

Considering the different kinetics and sensitivity of GVL response in hematologic malignancies enrolled in this study, the relapse and survival could not have been compared with other reports. In the current study, a total of six patients with a disease in NR status prior to transplantation relapsed at a median of 114 days after DLI, and the statistical analysis revealed that disease in NR status prior to transplantation was an independent risk factor for relapse after DLI. Reasons for the treatment failure might be associated with the poor GVL effect of DLI. The high proliferative kinetics of leukemia cells is one cause for poor GVL effect of DLI. Nevertheless, the immune evasion of leukemia cells by mechanisms such as loss of the patient-specific HLA haplotype represents another cause for poor GVL effect of DLI [27]. Therefore, it is likely the future of DLI should involve more strategies to enhance the GVL effect of the infused donor cells via using cytokines like interferon [28], selection or depletion of specific donor lymphocytes subsets [29, 30], genetically modified donor lymphocytes targeting of tumor-specific antigens, second prophylactic DLI in case of no GVHD occurrence, or combination with target therapy.

In summary, the data from this study suggested the tolerance and efficacy of prophylactic DLI in patients with very high-risk leukemia/lymphoma in the unmanipulated haplo-PBSCT setting with PB as grafts. Further study is required to determine kinetics of relapse in each subtype of high-risk malignancies and the optimal long-term prophylactic strategy.

## Electronic supplementary material

ESM 1 (DOCX 32 kb)

ESM 2 (DOCX 26 kb)

## References

[1] Schlenk RF, Döhner K, Mack S, Stoppel M, Király F, Götze K, Hartmann F, Horst HA, Koller E, Petzer A, Grimminger W, Kobbe G, Glasmacher A, Salwender H, Kirchen H, Haase D, Kremers S, Matzdorff A, Benner A, Döhner H (2010). Prospective evaluation of allogeneic hematopoietic stem-cell transplantation from matched related and matched unrelated donors in younger adults with high-risk acute myeloid leukemia: German-Austrian trial AMLHD98A. J Clin Oncol.

[2] Schmid C, Schleuning M, Ledderose G, Tischer J, Kolb HJ (2005). Sequential regimen of chemotherapy, reduced-intensity conditioning for allogeneic stem-cell transplantation, and prophylactic donor lymphocyte transfusion in high-risk acute myeloid leukemia and myelodysplastic syndrome. J Clin Oncol.

[3] Middeke JM, Herold S, Rücker-Braun E, Berdel WE, Stelljes M, Kaufmann M, Schäfer-Eckart K, Baldus CD, Stuhlmann R, Ho AD, Einsele H, Rösler W, Serve H, Hänel M, Sohlbach K, Klesse C, Mohr B, Heidenreich F, Stölzel F, Röllig C, Platzbecker U, Ehninger G, Bornhäuser M, Thiede C, Schetelig J, Study Alliance Leukaemia (SAL) (2016). TP53 mutation in patients with high-risk acute myeloid leukaemia treated with allogeneic haematopoietic stem cell transplantation. Br J Haematol.

[4] Hemmati PG, Vuong LG, Terwey TH, Jehn CF, le Coutre P, Penack O, Na IK, Dörken B, Arnold R (2017). Predictive significance of the European LeukemiaNet classification of genetic aberrations in patients with acute myeloid leukaemia undergoing allogeneic stem cell transplantation. Eur J Haematol.

[5] Ahn JS, Kim HJ, Kim YK, Jung SH, Yang DH, Lee JJ, Lee IK, Kim NY, Minden MD, Jung CW, Jang JH, Kim HJ, Moon JH, Sohn SK, Won JH, Kim SH, Kim N, Yoshida K, Ogawa S, Kim DD (2015). Adverse prognostic effect of homozygous TET2 mutation on the relapse risk of acute myeloid leukemia in patients of normal karyotype. Haematologica.

[6] Duval M, Klein JP, He W, Cahn JY, Cairo M, Camitta BM, Kamble R, Copelan E, de Lima M, Gupta V, Keating A, Lazarus HM, Litzow MR, Marks DI, Maziarz RT, Rizzieri DA, Schiller G, Schultz KR, Tallman MS, Weisdorf D (2010). Hematopoietic stem-cell transplantation for acute leukemia in relapse or primary induction failure. J Clin Oncol.

[7] Locke F, Agarwal R, Kunnavakkam R, van Besien K, Larson RA, Odenike O, Godley LA, Liu H, Le Beau MM, Gurbuxani S, Thirman MJ, Sipkins D, White C, Artz A, Stock W (2013). A novel clofarabine bridge strategy facilitates allogeneic transplantation in patients with relapsed/refractory leukemia and high-risk myelodysplastic syndromes. Bone Marrow Transplant.

[8] Liga M, Triantafyllou E, Tiniakou M, Lambropoulou P, Karakantza M, Zoumbos NC, Spyridonidis A (2013). High alloreactivity of low-dose prophylactic donor lymphocyte infusion in patients with acute leukemia undergoing allogeneic hematopoietic cell transplantation with an alemtuzumab-containing conditioning regimen. Biol Blood Marrow Transplant.

[9] Slavin S, Morecki S, Weiss L, Or R (2002). Donor lymphocyte infusion: the use of alloreactive and tumor-reactive lymphocytes for immunotherapy of malignant and nonmalignant diseases in conjunction with allogeneic stem cell transplantation. J Hematother Stem Cell Res.

[10] Jun HX, Jun CY, Yu ZX (2004). In vivo induction of T-cell hyporesponsiveness and alteration of immunological cells of bone marrow grafts using granulocyte colony-stimulating factor. Haematologica.

[11] Yan CH, Liu DH, Xu LP, Liu KY, Zhao T, Wang Y, Chen H, Chen YH, Han W, Huang XJ (2012). Modified donor lymphocyte infusion-associated acute graft-versus-host disease after haploidentical T-cell-replete hematopoietic stem cell transplantation: incidence and risk factors. Clin Transpl.

[12] Huang XJ, Liu DH, Liu KY, Xu LP, Chen H, Han W (2007). Donor lymphocyte infusion for the treatment of leukemia relapse after HLA-mismatched/haploidentical T-cell-replete hematopoietic stem cell transplantation. Haematologica.

[13] Huang XJ, Liu DH, Liu KY, Xu LP, Chen YH, Wang Y, Han W, Chen H (2008). Modified donor lymphocyte infusion after HLA-mismatched/haploidentical T cell-replete hematopoietic stem cell transplantation for prophylaxis of relapse of leukemia in patients with advanced leukemia. J Clin Immunol.

[14] Li HH, Li F, Gao CJ, Huang WR, Bo J, Dou LP, Wang LL, Jing Y, Wang L, Li WJ, Yu L, Liu DH (2017). Similar incidence of severe acute GVHD and less severe chronic GVHD in PBSCT from unmanipulated, haploidentical donors compared with that from matched sibling donors for patients with haematological malignancies. Br J Haematol.

[15] Rowlings PA, Przepiorka D, Klein JP, Gale RP, Passweg JR, Henslee-Downey PJ, Cahn JY, Calderwood S, Gratwohl A, Socié G, Abecasis MM, Sobocinski KA, Zhang MJ, Horowitz MM (1997). IBMTR severity index for grading acute graft-versus-host disease: retrospective comparison with Glucksberg grade. Br J Haematol.

[16] Jagasia MH, Greinix HT, Arora M, Williams KM, Wolff D, Cowen EW, Palmer J, Weisdorf D, Treister NS, Cheng GS, Kerr H, Stratton P, Duarte RF, McDonald GB, Inamoto Y, Vigorito A, Arai S, Datiles MB, Jacobsohn D, Heller T, Kitko CL, Mitchell SA, Martin PJ, Shulman H, Wu RS, Cutler CS, Vogelsang GB, Lee SJ, Pavletic SZ, Flowers ME (2015). National institutes of health consensus development project on criteria for clinical trials in chronic graft-versus-host disease: I. the 2014 diagnosis and staging working group report. Biol Blood Marrow Transplant.

[17] Scrucca L, Santucci A, Aversa F (2007). Competing risk analysis using R: an easy guide for clinicians. Bone Marrow Transplant.

[18] Scrucca L, Santucci A, Aversa F (2010). Regression modeling of competing risk using R: an in depth guide for clinicians. Bone Marrow Transplant.

[19] Huang XJ, Guo NL, Ren HY (2003). The comparison of GVL effects between the patients receiving donor peripheral blood stem cells and donor lymphocytes after allogeneic bone marrow transplantation. J Peking Univ Health Sci.

[20] Yan CH, Liu DH, Liu KY, Xu LP, Liu YR, Chen H, Han W, Wang Y, Qin YZ, Huang XJ (2012). Risk stratification-directed donor lymphocyte infusion could reduce relapse of standard-risk acute leukemia patients after allogeneic hematopoietic stem cell transplantation. Blood.

[21] Huang XJ, Chang YJ, Zhao XY (2007). Maintaining hyporesponsiveness and polarization potential of T cells after in vitro mixture of G-CSF mobilized peripheral blood grafts and G-CSF primed bone marrow grafts in different proportions. Transpl Immunol.

[22] Morris ES, MacDonald KP, Rowe V, Banovic T, Kuns RD, Don AL, Bofinger HM, Burman AC, Olver SD, Kienzle N, Porcelli SA, Pellicci DG, Godfrey DI, Smyth MJ, Hill GR (2005). NKT cell-dependent leukemia eradication following stem cell mobilization with potent G-CSF analogs. J Clin Invest.

[23] Friedrichs B, Tichelli A, Bacigalupo A, Russell NH, Ruutu T, Shapira MY, Beksac M, Hasenclever D, Socié G, Schmitz N (2010). Long-term outcome and late effects in patients transplanted with mobilized blood or bone marrow: a randomized trial. Lancet Oncol.

[24] Hasskarl J, Zerweck A, Wäsch R, Ihorst G, Bertz H, Finke J (2012). Induction of graft versus malignancy effect after unrelated allogeneic PBSCT using donor lymphocyte infusions derived from frozen aliquots of the original graft. Bone Marrow Transplant.

[25] Wang Y, Liu DH, Xu LP, Liu KY, Chen H, Zhang XH, Chen YH, Han W, Wang FR, Wang JZ, Yan CH, Huang XJ (2012). Prevention of relapse using granulocyte CSF-primed PBPCs following HLA-mismatched/haploidentical, T-cell-replete hematopoietic SCT in patients with advanced-stage acute leukemia: a retrospective risk-factor analysis. Bone Marrow Transplant.

[26] Inoue Y, Fuji, Tanosaki, Inamoto Y, Tanaka T, Ito A, Okinaka K, Kurosawa S, Kim SW, Nakagama H, Fukuda T (2018) Prognostic importance of pretransplant disease status for posttransplant outcomes in patients with adult T cell leukemia/lymphoma. Bone Marrow Transplant. 10.1038/s41409-018-0139-z 10.1038/s41409-018-0139-z PMC7102069 29523883

[27] Vago L, Perna SK, Zanussi M, Mazzi B, Barlassina C, Stanghellini MT, Perrelli NF, Cosentino C, Torri F, Angius A, Forno B, Casucci M, Bernardi M, Peccatori J, Corti C, Bondanza A, Ferrari M, Rossini S, Roncarolo MG, Bordignon C, Bonini C, Ciceri F, Fleischhauer K (2009). Loss of mismatched HLA in leukemia after stem-cell transplantation. N Engl J Med.

[28] Gesundheit B, Shapira MY, Resnick IB, Amar A, Kristt D, Dray L, Budowski E, Or R (2009). Successful cell-mediated cytokine-activated immunotherapy for relapsed acute myeloid leukemia after hematopoietic stem cell transplantation. Am J Hematol.

[29] Meyer RG, Britten CM, Wehler D, Bender K, Hess G, Konur A, Hartwig UF, Wehler TC, Ullmann AJ, Gentilini C, Uharek L, Huber C, Kolbe K, Herr W (2007). Prophylactic transfer of CD8-depleted donor lymphocytes after T-cell-depleted reduced-intensity transplantation. Blood.

[30] Dodero A, Carniti C, Raganato A, Vendramin A, Farina L, Spina F, Carlo-Stella C, Di Terlizzi S, Milanesi M, Longoni P, Gandola L, Lombardo C, Corradini P (2009). Haploidentical stem cell transplantation after a reduced-intensity conditioning regimen for the treatment of advanced hematologic malignancies: posttransplantation CD8-depleted donor lymphocyte infusions contribute to improve T-cell recovery. Blood.

