# Supplementary material for: Donor lymphocyte infusion for prevention of relapse after unmanipulated haploidentical PBSCT for very high-risk hematologic malignancies
Source: Ann Hematol. 2018 Aug 24;98(1):185–93. doi: 10.1007/s00277-018-3482-7 (PMC6334751; doi:10.1007/s00277-018-3482-7)
Supplement: Supplementary file 1 — (DOCX 32 kb) [file 277_2018_3482_MOESM1_ESM.docx]

Table 1 Pre-transplant recipient and donor characteristics of the very high-risk patients.

| Patient No. | Age | Gender | Disease status prior to HCT | WBC at diagnosis (10^9^/L) | Gene mutation | Cytogenetics | Age of donor | HLA matched loci | Donor | Blood type (Donor-recipient) | MNCs (10^8^/kg) | CD34^+^ (10^6^/kg) |
| --- | --- | --- | --- | --- | --- | --- | --- | --- | --- | --- | --- | --- |
| 1 | 23 | M | CML, CP1 | 4.37 | BCR/ABL T315I | 46, XY, t(9;22)(q34;q11) | 32 | 5/10 | Sister | B-B | 6.10 | 4.22 |
| 2 | 47 | M | PCL, CR1 after 3 courses of induction | N/A | TP53 | CK | 23 | 6/10 | Daughter | A-AB | 7.73 | 3.43 |
| 3 | 39 | M | B-ALL, CR1 | 3.1 | TP53 | FISH: -17 | 17 | 5/10 | Son | B-O | 6.31 | 6.37 |
| 4 | 20 | M | B-ALL, CR1 after 6 courses of induction | 17.44 | None | 46, XY, ?del(7)(p21) | 46 | 8/10 | Mother | B-B | 22.87 | 2.17 |
| 5 | 37 | M | AML-M4, CR1 | 9.02 | TET2 | 47, XY, +9 | 36 | 5/10 | Brother | A-A | 11.61 | 3.24 |
| 6 | 46 | F | AML-M5, CR1 | 14.99 | FLT3-ITD, NPM1, DNMT3a | 46, XX | 21 | 5/10 | Daughter | AB-A | 9.94 | 1.91 |
| 7 | 53 | F | AML-M2, NR | N/A | None | 46, XX, t(8;21)(q22;q22) | 25 | 5/10 | Son | B-B | 9.71 | 2.58 |
| 8 | 29 | F | AML-M5, NR | 49.9 | MLL/ENL | 46, XX | 52 | 5/10 | Father | A-A | 10.78 | 3.30 |
| 9 | 51 | F | Cytotoxic T-Cell Lymphoma, NR | N/A | None | 46, XX | 49 | 7/10 | Sister | A-O | 13.65 | 3.50 |
| 10 | 19 | M | AML-M2, RL | 4.29 | MLL/AF9, HOX11, FLT3-ITD, KIT, GATA-2, TET2, ASXL1 | 47, XY, +8 | 22 | 5/10 | Cousin | O-B | 6.22 | 3.36 |
| 11 | 42 | M | CML-CP1 | 2.31 | BCR/ABL T315I | 46, XY, t(9;22) | 24 | 5/10 | Daughter | A-AB | 7.20 | 4.05 |
| 12 | 28 | M | AML, CR1 | 16.65 | TET2 | 46, XY | 53 | 5/10 | Father | A-AB | 8.63 | 7.55 |
| 13 | 50 | M | AML-M5, CR1 | 67.8 | NPM1, SMC3, FLT3-ITD | 46, XX | 24 | 5/10 | Son | A-B | 6.43 | 4.75 |
| 14 | 19 | M | AML-M5, CR1 after 3 courses of induction | 2 | None | 46, XY | 45 | 5/10 | Father | B-B | 14.30 | 6.77 |
| 15 | 22 | M | AML-M2, CR1 | 10.2 | TP53, FLT3-TKD, CSF3R, SRSF2, | 45, X, -Y, t(8;21)(q22;q22) | 23 | 5/10 | Sister | O-O | 7.15 | 4.50 |
| 16 | 32 | M | AML-M1, CR1 | 35.23 | FLT3-ITD | N/D | 35 | 5/10 | Sister | O-O | 8.13 | 2.34 |
| 17 | 24 | M | T-LBL, CR1 | 55 | TP53, SET/CAN | 46, XY | 22 | 5/10 | Brother | AB-A | 5.47 | 5.76 |
| 18 | 48 | F | AML-M5, CR1 | 45 | DNMT3a, FLT3-ITD, NPM1, FLT3-TKD, CBL | 46, XX | 25 | 5/10 | Son | O-A | 11.04 | 6.29 |
| 19 | 52 | F | AML, CR1 after 3 courses of induction | 6.75 | None | 46, XX | 27 | 5/10 | Son | A-O | 13.45 | 4.25 |
| 20 | 27 | F | AML-M2, CR1 | 6.56 | FLT3-ITD | N/D | 33 | 5/10 | Sister | A-A | 11.87 | 5.92 |
| 21 | 20 | M | MDS-AML, NR | 3.47 | None | 46, XY | 28 | 5/10 | Cousin | B-O | 8.56 | 7.12 |
| 22 | 50 | F | T-LBL, NR | 2.17 | TP53, WT1 | 46, XX | 24 | 5/10 | Daughter | B-O | 7.70 | 6.83 |
| 23 | 34 | F | AML, CR1 | 13.5 | TET2, CEBPA | 46, XX, i(21)(q10) | 30 | 6/10 | Brother | B-B | 14.93 | 2.30 |
| 24 | 39 | F | AML-M2, RL | N/A | NPM1, WT1 | 46, XX | 31 | 5/10 | Cousin | O-A | 10.40 | 6.87 |
| 25 | 26 | F | MDS-AML, NR | 44 | DNMT3a, NRAS, KRAS, GATA2 | 46,XX | 19 | 5/10 | Brother | O-A | 8.89 | 4.26 |
| 26 | 29 | F | AML-M2, RL | 3.03 | TET2, RUNX1 | 46,XX, t(8;21)(q22;q22) | 55 | 5/10 | Father | A-A | 14.84 | 4.40 |
| 27 | 54 | M | B-ALL, CR1 | 7.65 | TP53 | 46,XY | 28 | 5/10 | Son | O-O | 6.39 | 5.87 |
| 28 | 30 | M | AML-M2, CR1 | N/A | TET2, KIT | 46,XY, t(8;21)(q22;q22) | 36 | 5/10 | Sister | A-A | 10.34 | 4.40 |
| 29 | 26 | M | AML-M2 | 8.11 | FLT3-TKD, KIT, NOTCH2, FAT1, IL7R | 46,XY | 54 | 5/10 | Father | O-A | 12.08 | 2.17 |
| 30 | 57 | F | B-ALL, CR1 | 186 | None | 46, XX | 26 | 6/10 | Son | AB-A | 8.46 | 3.15 |
| 31 | 18 | M | AML-M5, CR1 | 10.99 | TET2, NARS, ASXL1, KMT2A | 47,XY,+10 | 50 | 5/10 | Father | B-AB | 5.2 | 3.00 |
| 32 | 15 | F | CML-CP2 | 70 | FLT3-ITD | 45, XX, -18 | 36 | 7/10 | Father | AB-A | 7.40 | 6.40 |
| 33 | 34 | F | AML-M2, CR1 after 5 courses of induction | 1.91 | None | CK | 32 | 5/10 | Brother | B-B | 8.66 | 9.08 |
| 34 | 31 | M | Cutaneous T-cell lymphoma, stage IV, NR | N/A | None | 46, XY | 34 | 5/10 | Sister | O-A | 8.75 | 10.80 |
| 35 | 7 | M | T-LBL, NR | N/A | None | N/D | 38 | 5/10 | Father | O-B | 11.20 | 6.57 |
| 36 | 61 | M | MDS-AML, NR | 0.64 | None | 47, XY, +8 | 58 | 5/10 | Brother | A-A | 9.48 | 3.75 |
| 37 | 46 | M | AML-M5, CR2 after 3 courses of induction | 4.73 | None | 46, XY | 22 | 5/10 | Son | A-A | 16.32 | 6.76 |
| 38 | 18 | M | T-LBL, PD | N/A | None | N/D | 45 | 5/10 | Father | A-A | 8.43 | 2.30 |
| 39 | 43 | M | AML-M0, RL | 3.6 | TP53 | CK | 49 | 5/10 | Sister | O-A | 7.66 | 3.95 |
| 40 | 32 | F | CML-BC | 41.3 | BCR/ABL T315I | 46, XX, t(9;22)(q34;p11) | 31 | 5/10 | Cousin | A-O | 9.47 | 10.98 |
| 41 | 39 | M | B-ALL, RL2 | 137.86 | BCR/ABL T315I | CK | 42 | 5/10 | Sister | AB-B | 8.42 | 7.96 |
| 42 | 53 | M | AML-M2, CR1 | 9.97 | None | 46, XY | 48 | 5/10 | Sister | A-B | 8.25 | 5.36 |
| 43 | 48 | M | AML-M4, CR1 | 76.2 | NPM1, NRAS, CUX1, TET2, RUNX1, SRSF2 | 46, XY | 22 | 5/10 | Daughter | AB-B | 8.30 | 2.44 |
| 44 | 33 | F | AML-M5, NR | 38.32 | NPM1, FLT3-TKD, DNMT3a, RUNX1, IDH1, SRSF2 | 46, XX | 66 | 5/10 | Father | O-B | 13.69 | 1.06 |
| 45 | 18 | M | T-LBL, NR | 3.12 | Bcl2 | 45, XY, del(11)(q14) | 23 | 5/10 | Sister | O-O | 6.15 | 6.27 |

ALL, acute lymphoblastic leukemia; AML, acutemyeolgenous leukemia; CML-CP, chronic myeloid leukemia in chronic phase; CML-BC, chronic myelogenous leukemia in blast crisis; CNS-L, central nervous system leukemia; CK, Complex karyotype; CR, complete remission; MDS-AML, AML transformed from myelodysplastic syndromes; MNCs, mononuclear cells; NR, non-remission; PCL, Plasma cell leukemia; RL, relapse; SD, stable disease; T-LBL, T-Cell lymphoblastic leukemia/lymphoma; WBC, white blood cell.
