# Supplementary material for: Donor lymphocyte infusion for prevention of relapse after unmanipulated haploidentical PBSCT for very high-risk hematologic malignancies
Source: Ann Hematol. 2018 Aug 24;98(1):185–93. doi: 10.1007/s00277-018-3482-7 (PMC6334751; doi:10.1007/s00277-018-3482-7)
Supplement: Supplementary file 2 — (DOCX 26 kb) [file 277_2018_3482_MOESM2_ESM.docx]

Table 2 Transplantation outcomes of the very high-risk patients.

| Patient No. | Neutrophil engraftment | Platelet engraftment | Time of DLI | Acute GVHD before DLI | Acute GVHD after DLI/HCT | Chronic GVHD after DLI/HCT | Outcomes |
| --- | --- | --- | --- | --- | --- | --- | --- |
| 1 | day +12 | day +14 | day +176 | None | None | Severe, day +300 after DLI | Died of GVHD and abandon of treatment on day +656 after HCT (day +480 after DLI) |
| 2 | day +10 | day +12 | day +84 | Grade 2, day +57 | Grade 2, day +72 after DLI | None | Died of viral pneumonia on day +217 after HCT (day +133 after DLI) |
| 3 | day +16 | day +25 | day +98 | None | None | None | Relapsed on day +174 after HCT (day +76 after DLI) |
| 4 | day +21 | day +36 | day +69 | None | None | moderate, day +168 after DLI | Relapsed on day +169 after HCT (day +100 after DLI) |
| 5 | day +15 | day +15 | day +116 | Grade 2, day +34 | Grade 2, day +40 after DLI | None | Died of intracranial hemorrhage on day +184 after HCT (day +68 after DLI) |
| 6 | day +11 | day +22 | day +96 | None | Grade 2, day +30 after DLI | Moderate, day +200 after DLI | Died of GVHD and abandon of treatment on day +345 after HCT (day +249 after DLI) |
| 7 | day +16 | day +18 | day +64 | Grade 1, day +25 | Grade 2, day +20 after DLI | Severe, day +60 after DLI | Died of chronic GVHD and pneumonia on day +209 after HCT (day +145 after DLI) |
| 8 | day +12 | day +13 | day +61 | None | None | None | Relapsed on day +240 after HCT (day +179 after DLI) |
| 9 | day +19 | day +39 | day +71 | None | None | None | Relapsed on day +96 after HCT (day +25 after DLI) |
| 10 | day +10 | day +12 | day +126 | Grade 2, day +40 after HCT | Grade 2, day +30 after DLI | Moderate, day +68 after DLI | Relapsed on day +194 after HCT (day +68 after DLI) |
| 11 | day +12 | day +19 | day +71 | None | None | None | RFS for 639 days after HCT (day +568 after DLI) |
| 12 | day +12 | day +13 | day +113 | Grade 2, day +28 after HCT | Grade 4, day +90 after DLI | Mild, day +215 after DLI | RFS for 387 days after HCT (day +274 after DLI) |
| 13 | day +11 | day +12 | day +71 | Grade 1, day +18 after HCT | Grade 2, day +28 after DLI | Severe, day +250 after DLI | Died of GVHD on day +321 after HCT (day +250 after DLI) |
| 14 | day +11 | day +10 | day +86 | Grade 2, day +46 after HCT | Grade 2, day +90 after DLI | Moderate, day +210 after DLI | RFS for 355 days after HCT (day +269 after DLI) |
| 15 | day +12 | day +15 | day +67 | Grade 2, day +37 after HCT | Grade 2, day +165 after DLI | None | RFS for 446 days after HCT (day +379 after DLI) |
| 16 | day +11 | day +19 | day +99 | Grade 2, day +39 after HCT | Grade 2, day +68 after DLI | None | RFS for 336 days after HCT (day +237 after DLI) |
| 17 | day +16 | day +21 | day +46 | None | Grade 2, day +75 after DLI | None | Relapsed on day +133 after HCT (day +87 after DLI) |
| 18 | day +10 | day +12 | day +56 | None | Grade 2, day +21 after DLI | None | RFS for 310 days after HCT (259 days after DLI) |
| 19 | day +10 | day +13 | day +45 | Grade 2, day +26 after HCT | None | None | RFS for 1174 days after HCT (1129 days after DLI) |
| 20 | day +10 | day +10 | day +93 | Grade 1, day +22 after HCT | None | None | RFS for 733 days after HCT (640 days after DLI) |
| 21 | day +10 | day +10 | day +109 | None | Grade 1, day +11 after DLI | Mild, day +582 after DLI | RFS for 750 days after HCT (641 days after DLI) |
| 22 | day +14 | day +19 | day +47 | Grade 1, day +30 after HCT | Grade 1, day +75 after DLI | Moderate, day +249 after DLI | Relapsed on day +379 after HCT (day +332 after DLI) |
| 23 | day +10 | day +14 | day +240 | Grade 4, day +31 after HCT; Grade 2, day +71 after HCT | None | None | RFS for 383 days after HCT (143 days after DLI) |
| 24 | day +11 | day +11 | day +99 | Grade 1, day +40 after HCT | Grade 4, day +100 after DLI | Moderate, day +120 after DLI | Relapsed on day +351 after HCT (day +252 after DLI) |
| 25 | day +10 | day +11 | day +60 | None | Grade 2, day +12 after DLI | None | RFS for 340 days after HCT (day +284 after DLI) |
| 26 | day +9 | day +9 | day +70 | Grade 1, day +56 after HCT | Grade 2, day +59 after DLI | None | RFS for 301 days after HCT (day +231 after DLI) |
| 27 | day +12 | day +21 | day +84 | Grade 2, day +44 after HCT | None | Severe, day +114 after DLI | Died of GVHD on day +239 after HCT (day +155 after DLI) |
| 28 | day +11 | day +14 | day +68 | Grade 1, day +24 after HCT | None | None | RFS for 242 days after HCT (day +174 after DLI) |
| 29 | day +14 | day +14 | day +101 | None | None | None | Relapsed on day +112 after HCT (day +11 after DLI) |
| 30 | day +13 | day +24 | day +77 | None | None | None | Died of pneumonia on day +133 after HCT (day +56 after DLI) |
| 31 | day +12 | day +17 | day +70 | Grade 2, day +34 after HCT | None | None | RFS for 111 days after HCT (day +41 after DLI) |
| 32 | day +10 | day +10 | - | - | Grade 2, day +25 after HCT | None | Relapsed after control of GVHD on day +92 after HCT |
| 33 | day +16 | day +19 | - | - | Grade 1, day +26 after HCT | Severe, day +168 after HCT | Relapsed after control of GVHD on day +168 after HCT |
| 34 | day +11 | day +15 | - | - | Grade 1, day +43 after HCT | None | Relapsed after control of GVHD on day +66 after HCT |
| 35 | day +11 | day +13 | - | - | Grade 2, day +28 after HCT | None | NR on day +35 after HCT |
| 36 | day +13 | day +16 | - | - | None | None | Relapsed on day +90 after HCT |
| 37 | day +13 | day +16 | - | - | Grade 4, day +46 after HCT | None | Died of GVHD on day +83 after HCT |
| 38 | day +15 | day +16 | - | - | None | None | NR after HCT on day +39 |
| 39 | day +19 | day +17 | - | - | Grade 2, day +42 after HCT | Moderate, day +120 after HCT | Died of GVHD on day +155 |
| 40 | day +15 | day +33 | - | - | Grade 4, day +77 after HCT | None | Relapsed after control of GVHD on day +264 after HCT |
| 41 | day +9 | day +53 | - | - | None | Mild, day +120 after HCT | Prophylaxis with Ponatinib and relapsed on day +77 |
| 42 | day +15 | day +20 | - | - | Grade 2, day +81 after HCT | None | RFS for 352 days after HCT |
| 43 | day +15 | day +52 | - | - | Grade 1, day +39 after HCT | None | RFS for 508 days after HCT |
| 44 | day +10 | day +120 | - | - | None | None | RFS for 1048 days after HCT |
| 45 | day +12 | day +14 | - | - | Grade 1, day +43 after HCT | None | RFS for 383 days after HCT |

CsA, cyclosporine; DLI, donor lymphocyte infusion; GVHD, graft-versus-host disease; HCT, hematopoietic stem cell transplantation; MRD, minimal residual disease; MTX, methotrexate; NR, non-remission; RFS, relapse-free survival.
